# Wearing a Wetsuit Alters Upper Extremity Motion during Simulated Surfboard Paddling

**DOI:** 10.1371/journal.pone.0142325

**Published:** 2015-11-09

**Authors:** J. A. Nessler, M. Silvas, S. Carpenter, S. C. Newcomer

**Affiliations:** 1 Department of Kinesiology, California State University, San Marcos, CA, United States of America; 2 Water’s Edge Physical Therapy and Wellness, Oceanside, CA, United States of America; Tianjin University, CHINA

## Abstract

Surfers often wear wetsuits while paddling in the ocean. This neoprene covering may be beneficial to upper extremity movement by helping to improve proprioceptive acuity, or it may be detrimental by providing increased resistance. The purpose of this study was to evaluate the effects of wearing a wetsuit on muscle activation, upper extremity motion, heart rate, and oxygen consumption during simulated surfboard paddling in the laboratory. Twelve male, recreational surfers performed two paddling trials at a constant workload on a swim bench ergometer both with and without a wetsuit. Kinematic data and EMG were acquired from the right arm via motion capture, and oxygen consumption and heart rate were recorded with a metabolic cart and heart rate monitor. Wearing a wetsuit had no significant effect on oxygen consumption or heart rate. A significant increase in EMG activation was observed for the middle deltoid but not for any of the other shoulder muscle evaluated. Finally, approximate entropy and estimates of the maximum Lyapunov exponent increased significantly for vertical trajectory of the right wrist (i.e. stroke height) when a wetsuit was worn. These results suggest that a 2mm wetsuit has little effect on the energy cost of paddling at lower workloads but does affect arm motion. These changes may be the result of enhanced proprioceptive acuity due to mechanical compression from the wetsuit.

## Introduction

Wetsuits have become an important piece of surfing equipment. They are primarily utilized to aid in thermoregulation for athletes performing in cooler water temperatures [1–3], but research indicates that wetsuits have additional effects on human performance. For example, wearing a wetsuit has been shown to improve swimming speed and energy efficiency in triathletes by increasing buoyancy and decreasing drag [4] [5]. In addition, the compressive effect of a wetsuit has been shown to lead to changes in cardiovascular behavior [6] and decreases in water retention and blood volume [7]. While it is unclear how these effects might benefit a surfing athlete, the anecdotal observation that many surfers prefer to wear wetsuits even when their thermoregulatory assistance is unnecessary (i.e. water temperatures exceeding 75°F) suggests that there may be additional benefits to performance.

One such wetsuit-related benefit might be associated with the biomechanics and control of arm motion during repetitive paddling. From one perspective, neoprene may provide resistance to arm motion, which may lead to increases in muscle activation and metabolic cost. Wearing restrictive clothing has been shown to have a similar effect during other forms of physical activity [8,9]. However, wearing a wetsuit might also affect arm motion through alterations in proprioceptive feedback. Neoprene sleeves covering individual joints are often used by athletes to improve performance, and many investigators have demonstrated that wearing neoprene sleeves can improve joint position sense in both the knee [10–12] and shoulder [13]. While supporting data is relatively sparse, it is generally thought that neoprene sleeves can enhance proprioception by stimulating mechanoreceptors, thereby leading to improved control and stability of a joint.

To date, evaluation of neoprene sleeves has focused primarily on changes in passive joint position sensing [10–13]. While these data provide evidence that a neoprene sleeve may contribute to enhanced proprioceptive acuity, they were recorded during isolated, single-joint movements, often in situations where subjects were not required to move under their own effort. Functional movements like surfboard paddling are inherently more complex because they require coordinated interaction of multiple segments, careful activation of bi-articular muscles, and voluntary planning and execution of the movement, as opposed to movement caused by external manipulation. Since the paddling stroke is complex, and a wetsuit could theoretically influence proprioceptive acuity across multiple joints in the upper extremity, it is unclear how a neoprene sleeve covering the entire arm might contribute to paddling performance. Further, active, volitional motion has been shown to generate feedback that is inherently more noisy and prone to error than the passive motion paradigms commonly used to measure joint position sense [14]. Additional research is needed to determine whether neoprene sleeves can alter the volitional control of larger, more complex movements, and whether such an effect might be achieved by wearing a wetsuit.

The study of surfboard paddling may provide insight to these questions. Neuromuscular control of repetitive movements like the paddling stroke can be evaluated by examining patterns of motion that occur over time using nonlinear analysis techniques [15–18]. These approaches assume that successive movement cycles are interdependent and that variability between cycles is a rich source of information about the nature of movement coordination. This type of analysis yields information regarding the underlying structure or organization of movement variability, rather than the average magnitude of variability, as is provided by standard deviation or coefficient of variance. Therefore, evaluating the relationships among stroke-to-stroke deviations in motion can be useful for understanding control throughout the entire paddling behavior. Some approaches to nonlinear analysis of time varying systems are based upon complex network theory [19–25]. Examples of these include directed weighted complex network analysis (DWCN) [20] and recurrence quantification analysis [25]. These techniques are sometimes preferred because they are more robust to noise in the system. Other techniques are based upon nonlinear dynamic system theory [15–18,26,27]. These approaches have been utilized most often in previous studies of human movement. Examples of these include estimates of maximal Lyapunov exponents (LyE) [17,18], Approximate Entropy (ApEn) [16], and detrended fluctuation analysis (DFA) [15,28].

Lyapunov exponents provide a measure of the rate of divergence over time of nearby trajectories in state space. In essence, Lyapunov exponents provide a measure of the sensitivity of a system to small perturbations, such as variations in stroke to stroke motion that occur naturally with repetitive motion such as surfboard paddling. This behavior is often referred to as the local dynamic stability of a time series [26,29]. In some situations, estimates of maximum Lyapunov exponents are used to infer the amount of divergence from a behavioral attractor [30]. However, while Lyapunov exponents can provide insight into certain aspects of the control of movement, the relationship between this statistic and skilled human motion is somewhat unclear. In some cases higher LyEs appear to be indicative of a novice learner or impaired neuromuscular control [27,31]. In other cases, lower values have been associated with peripheral neuropathy [26] or musculoskeletal injury [32]. This complex relationship has lead researchers to propose the idea that there exists an optimal, chaotic structure to movement variability [33]. Low values of LyE are associated with a more predictable, rigid, and inflexible system that is limited in its ability to adapt to changing conditions and whose lack of appropriate variability in repetitive movements may place an individual at greater risk for overuse injury [26,32]. Conversely, greater values of LyE are associated with a noisy, random, and unpredictable system that is often associated with older adults and developing infants [27,31]. Both of these extremes have been related with either a lack of movement skill or reduced health, and the ideal behavior lies somewhere in between.

Approximate Entropy (ApEn) provides a measure of complexity or irregularity in a system by estimating the probability that non-repeating patterns of data that begin in close proximity will remain close when they are incremented forward by one step in the time series. Values of ApEn that are close to 0 suggest that a system is highly regular, while values close to 2 suggest that a system is highly irregular or complex. As an example, a periodic sine wave will exhibit a value of ApEn close to 0.17, while the Lorenz attractor, a chaotic system, typically exhibits a value of ApEn close to 0.5 [34]. When studying human movement, a lower level of complexity is often associated with increased age [35], disease [36], and injury [37]. Higher levels of complexity have been associated with greater skill or proficiency in human movement [38,39].

Detrended fluctuation analysis (DFA) provides an approach for quantifying statistical persistence, or how closely the characteristics of a particular stroke are related to those of previous and subsequent strokes [40,41]. While understanding the statistical persistence of the paddling stroke would be useful, the reliability of DFA has been shown to be sensitive to the length of the time series data [42]. Paddling trials of sufficient length (approximately 600 continuous strokes) would be overly taxing for surfers and therefore DFA was not performed in the current study.

The purpose of this study was to investigate the effect of wearing a wetsuit on paddling motion in surfers. Two questions were addressed: 1) For a constant paddling velocity, does wearing a wetsuit have an effect on muscle activation and energy use, and 2) does wearing a wetsuit have an effect on the control of repetitive arm motion? Because previous literature has reported that certain types of restrictive clothing can lead to increases in EMG and energy use [8,9], it was hypothesized that wearing a wetsuit would have a similar effect. Further, because previous literature has reported that proprioception can be enhanced while wearing neoprene sleeves [10–13], it was also hypothesized that wearing a wetsuit would result in changes in the control of arm motion, manifest as differences in arm kinematics, ApEn and LyE. Portions of these data have been published in conference paper format [43].

## Methods

### Subjects

Twelve male, recreational surfers were recruited from the local surfing population (Table 1). Each participant indicated that they engaged in surfing at least 8 hours per week on average and that surfing was their primary form of exercise. All subjects completed a health history questionnaire (AHA/ACSM Health/Fitness Facility Participation Screening Questionnaire) and shoulder range of motion screen, which included assessment of motion in the sagittal and frontal planes using standard goniometric techniques [44] and Apley’s scratch test [45]. Subjects that did not meet normative levels of shoulder mobility and symmetry [44,45], or those that reported pain during the shoulder screen did not participate. All subjects were free of any cardiovascular, musculoskeletal, or neurological condition that might have affected performance. All procedures were approved by the Institutional Review Board at California State University, San Marcos (IRB# 2014–096), and all participants gave their written informed consent prior to participation.

**Table 1:** Subject Characteristics (n = 12).

	Age [years]	Height [m]	Mass [kg]	Surfing Experience [years]
**Mean**	33.1±8.6	1.82±0.07	79.9±9.4	21.8±12.4
**Range**	19–43	1.73–1.96	64.8–91.5	2–38

Values are reported as mean±SD

### General Procedures

Each participant paddled at a submaximal level for 5 minutes while wearing a wetsuit and 5 minutes without a wetsuit, with a 15 minute rest in between. For each trial, participants were given time to reach a physiological steady state during the first 3 minutes of paddling and data were collected during the final 2 minutes. The same model of front-zip, neoprene jacket (2 mm thickness) was utilized for all wetsuit trials. In order to avoid damage to the wireless EMG sensors, the jackets remained dry. The order in which each trial was performed was randomized across subjects.

**Fig 1:**
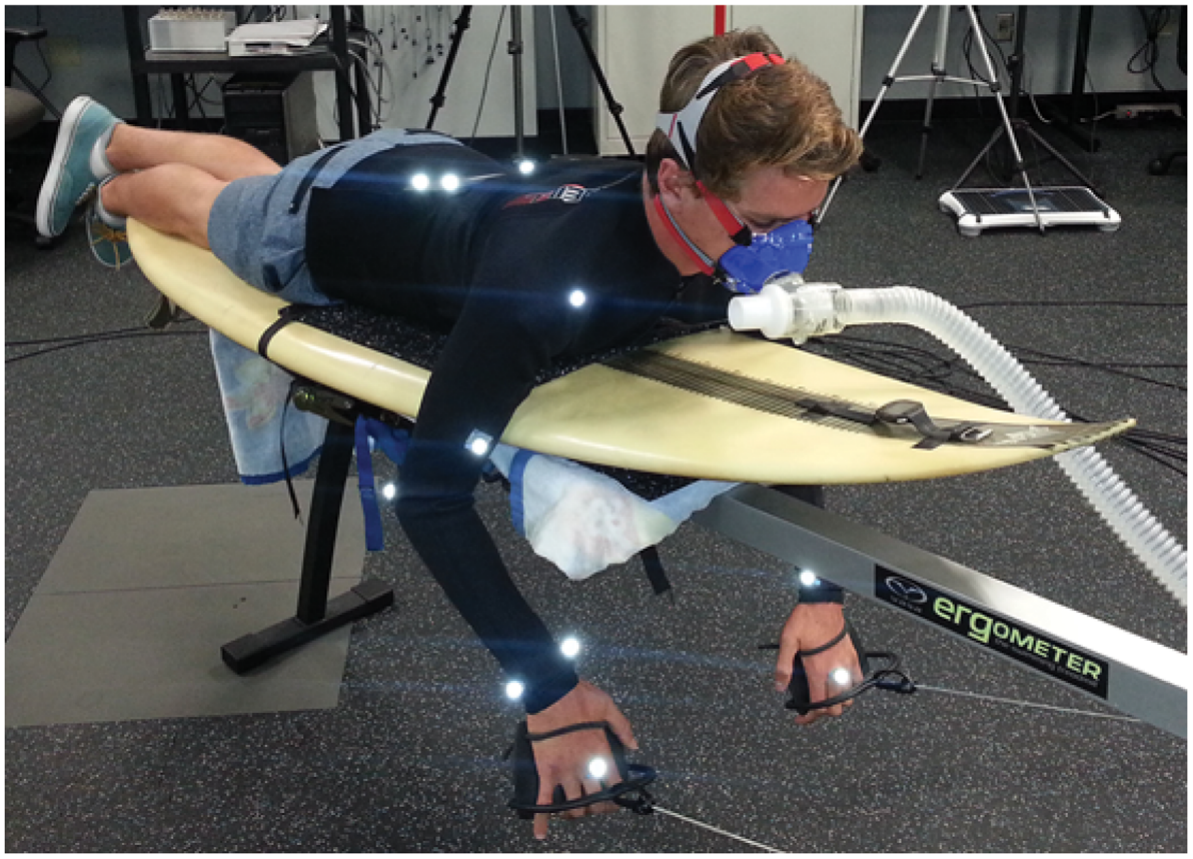
Experimental setup. Modified swim bench ergometer, marker placement, and VO_2_ mask.

Paddling was simulated in the laboratory using a commercially available swim bench ergometer (VASA Inc., Essex Junction, VT) that was modified by rigidly attaching a short surfboard to the top aspect of the bench (Fig 1). Participants’ hands were strapped to small paddles that were attached to a cable and pulley mechanism that provided resistance by spinning a small wind turbine that simulated water loads. Power generated by the subject was output in real time to a small digital screen. In order to ensure a consistent effort subjects were asked to maintain a power output as close to 20 Watts as possible for the duration of the trial. Subjects were also asked to match their paddling cadence to an audible metronome at 25 bpm. This rate and intensity of paddling was selected to approximate behavior when surfers paddle out from shore, where a sustained paddle at lower intensity is often utilized. Power output and strokes per minute were video recorded and analyzed offline to ensure subject adherence to these parameters. Stroke rate was also determined from motion capture data (details provided below). Trials with fluctuations greater than 2 standard deviations from the criterion values for stroke rate or power output were considered outliers and were not included in the final analysis. Arm movement was relatively unrestricted, though subjects were instructed to paddle in a manner that was similar to their normal motion while in water. This included lifting their hand above the level of the surfboard with each stroke to simulate bringing the hand out of the water. To ensure consistent placement of the subject on the apparatus between trials, a small grid was created on the top of the stationary surfboard.

Skin temperature was recorded each minute using a small, wireless iButton thermal sensor (type DS1921G; Maxim/Dallas Semiconductor Corp., USA) that was taped to the lateral aspect of the back at approximately T12. Oxygen consumption was measured at 15 second intervals with a Metabolic Measurement System (type TrueOne 2400; ParvoMedics Inc., USA). A heart rate monitor (type RCX5 receiver & T31 recorder, Polar, Finland) was strapped below the pectoralis major muscles of the subjects and heart rates were recorded at 5 second intervals.

An 8 camera Vicon motion capture system was used to track reflective markers placed over right arm of each subject at 120 Hz. Markers were placed over the head of the second metacarpal, the radial and ulnar styloid processes, the olecranon, the middle/lateral aspect of the upper arm, the acromion process, and on the lower back. While wearing a wetsuit, the neoprene over the surface of the shoulder can move significantly with respect to the skin and anatomical landmarks. Therefore, the acromion process marker was placed on a short post (approximately 1.5 cm) that was attached directly to the skin for both trials. During the wetsuit trials, this marker and post protruded through a small hole in the wetsuit. Surface EMG was utilized to record muscle activity from the medial head of the *triceps brachii*, *latissimus dorsi*, *infraspinatus*, *pectoralis major*, the upper and middle *trapezius*, middle *deltoid* and the lumbar region of *erector spinae* on the right hand side of the subject only. EMG data were captured at 960 Hz using a Delsys Trigno wireless system that was synchronized with the motion capture system.

### Data Analysis

Metabolic consumption, temperature, and heart rate data were analyzed off-line by finding the mean for each subject across each 2 minute trial. All other calculations were performed using custom routines written in MATLAB (R2014b, Natick, MA), the TISEAN software package (version 3.0.1) [46], or software created by Perc [47] (mutual information and false nearest neighbor calculations). The trajectory of the right wrist in the sagittal plane was utilized to define the beginning and ending of each stroke as the point of greatest anterior (or cranial) displacement of the hand. Stroke lengths, heights, widths, and durations, as well as shoulder range of motion in the sagittal and frontal planes were then calculated across all defined and complete strokes. These data were then analyzed for regularity and/or complexity using the Approximate Entropy approach (ApEn, described below) and for local dynamic stability by calculating maximal Lyapunov exponents (LyE). Detrended fluctuation analysis (DFA) was not utilized here as data files were considered too small to yield reliable results (approximately 50 strokes per trial) [42]. Offline, EMG data were first rectified and low-pass filtered (4^th^ order Butterworth, 40 Hz cutoff). Muscle activation was then parsed into each individual stroke as defined by wrist marker trajectory, and the average muscle activation profile was calculated for each muscle across all strokes. The peak level of activation, area under the EMG curve (linear envelope), and burst duration were then calculated for each muscle under both paddling conditions.

Approximate entropy (ApEn) was calculated using an approach previously applied to the analysis of gait and postural sway data [34,48]. This analysis began with a reduction of the original time series data by calculating relevant features from each stroke such as length, height, or duration. For example, the lengths of all strokes for the entire 2 minute wetsuit trial for a particular subject were combined to form a data set *X* of length *N*. In this case, *X*_*m*_ *(i)* = [*x(i)*, *x(i+1)*,*…*,*x(i+m-1)*] represents a vector segment of dimension *m* taken from the original data set *X* such that [*X*_*m*_ *(1)*, *X*_*m*_ *(2)*,*…*,*X*_*m*_ *(N-m+1)*] defines the complete set of vector segments. In the equations below, a tolerance region *r* was also identified and used to evaluate the distance between *X*_*m*_ *(i)* and *X*_*m*_ *(j)*. For the current analysis, these values were *m* = 2 and *r* = 0.25 x SD of *X*. These values were selected because previous analyses have reported that good statistical validity can be achieved for time series with lengths between 50 and 5000 data points by setting these parameters in this range [48,49]. In addition, previous analyses of human movement have also used similar values for m and r [50,51]. Consistent use of these settings will facilitate comparison with previous work. ApEn(*m*,*r*,*N*) was then calculated in three steps. First, $C_{i}^{m}$(r) can be defined as:

$$C_{i}^{m}\left(r\right)=\frac{1}{N-m}\Sigma_{j=1}^{N-m}\Theta \left(r-∥X_{m}\left(i\right)-X_{m}\left(j\right)∥\right)$$ (1)

where ||•|| denotes the distance between each X_m_(i) and X_m_(j) (calculated as the largest difference between respective scalar components) and Θ represents the Heaviside step function (Θ(s) = 0 if s<0 and Θ(s) = 1 if s≥1). Second, Φ^m^(r) can be calculated as:

$$\Phi^{m}\left(r\right)=\frac{1}{N-m}\Sigma_{j=1}^{N-m}\text{ln}C_{j}^{m}$$ (2)

and finally Approximate Entropy:

$$ApEn\left(m,r,N\right)=\Phi^{m}\left(r\right)-\Phi^{m+1}\left(r\right)$$ (3)

**Fig 2:**
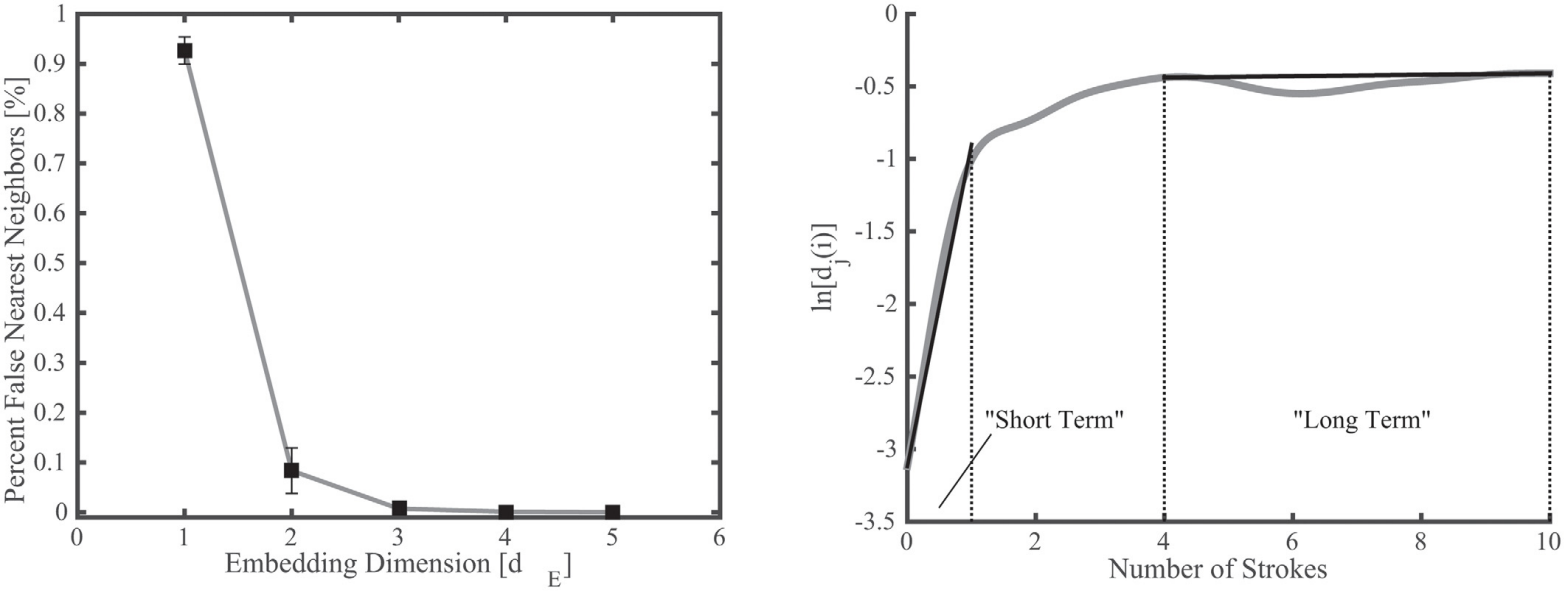
**Left: Percent false nearest neighbors for a range of embedding dimensions for wrist trajectory in the vertical direction.** An embedding dimension of 4 was utilized for calculation of maximal Lyapunov exponents. **Right: sample divergence curve used to calculate maximal Lyapunov exponents for a single subject.** Short term values were calculated over 0–1 stroke and long term values were calculated over 4–10 strokes.

Calculation of Lyapunov exponents for wrist trajectory in each of the 3 dimensions was performed using a combination of the TISEAN software package for estimation of divergence curves [46], and MATLAB routines for calculating the slopes of those curves. Pre-processing of data began with the reconstruction of the state space of each time series according to the model:

$$X\left(t\right)=\left[x\left(t\right),\;x\left(t+\tau \right),\ldots ,x(t+\left(d_{E}-1\right)\tau \right]$$ (4)

where *X*(*t*) represents the new state vector of dimension *d*_E_, which retains the properties of the original time series, *x*(*t*), with time delay *τ* and embedding dimension *d*_*E*_. There are multiple methods for estimating appropriate values for *d*_*E*_ and *τ* for time series data [52–56]. These methods do not always yield similar results [52,53]. Some of these techniques are based upon the assumption that *d*_*E*_ and *τ* are independent [29,55,56], while others assume that these two parameters are closely related [53,54,57]. Further, most techniques require the assignment of parameters such as tolerance regions which may also affect the outcome of the analysis. A detailed explanation of each approach is beyond the scope of this work, but the reader is directed to the references cited above. For the current data, the embedding delay (*τ*) was determined by finding the first minima of the average mutual information algorithm [29,55], and the embedding dimension (*d*_*E*_) was selected using results from the false nearest neighbor algorithm [56]. These approaches are used most frequently by researchers who study human movement (e.g. [26,27,30]), and consistency with previous work was sought here in order to facilitate comparison. While an embedding dimension of 5 or 6 has been used previously for repetitive motion of the ankle during gait [18,26,58], an embedding dimension of 4 (Fig 2) and embedding delay of 20 were selected for the paddling data analyzed here.

The true maximum Lyapunov exponent for a system is defined by:

$$d\left(t\right)=De^{\lambda t}$$ (5)

where D refers to the initial Euclidean distance between neighboring points, and d(t) refers to their average separation in state space at time t. Note that λ is only well defined as *D*→0 and *t*→∞. Since it is not possible to approach these limits with physiological time series data, an approximation for the finite-time, maximum Lyapunov exponent can be found by using the log-transform of eq 5. Divergence curves were created here using the method described by Rosenstein et al. [59]:

$$\text{ln}\left[d_{j}\left(i\right)\right]\approx \text{ln}D_{j}+\lambda^{*}\left(i\cdot \Delta t\right)$$ (6)

where *d*_*j*_ *(i)* refers to the Euclidean distance of the *j* ^th^ pair of nearest neighbors following *i* discrete time steps, and *D*_*j*_ refers to their initial Euclidean distance. The estimate of finite-time maximal Lyapunov exponents (λ*) over all pairs of nearest neighbors requires the calculation of a mean divergence curve (*y*) as follows:

$$y\left(i\right)=\left(\frac{1}{\Delta \text{t}}\right)\left[\text{ln}\;d_{j}\left(i\right)\right]$$ (7)

where […] represents the average over all pairs. Using average stroke duration, the time of each divergence curve was then normalized to number of strokes, and the slope was then calculated over both 0–1 stroke (“short-term”, λ*_short_) and 4–10 strokes (“long-term”, λ*_long_) (Fig 2). This approach is similar to that used in the analysis of ankle trajectory during gait (i.e. 0–1 stride and 4–10 strides) [18,26].

Paired t-tests were used to compare differences between wetsuit/no-wetsuit conditions for each variable (α = 0.05). Though multiple comparisons can increase the probability for type I error, statistical correction may not be appropriate for some of the variables included here as they may not be independent of one another. Therefore, all p-values below 0.05 are highlighted below.

## Results

### Arm Kinematics

**Fig 3:**
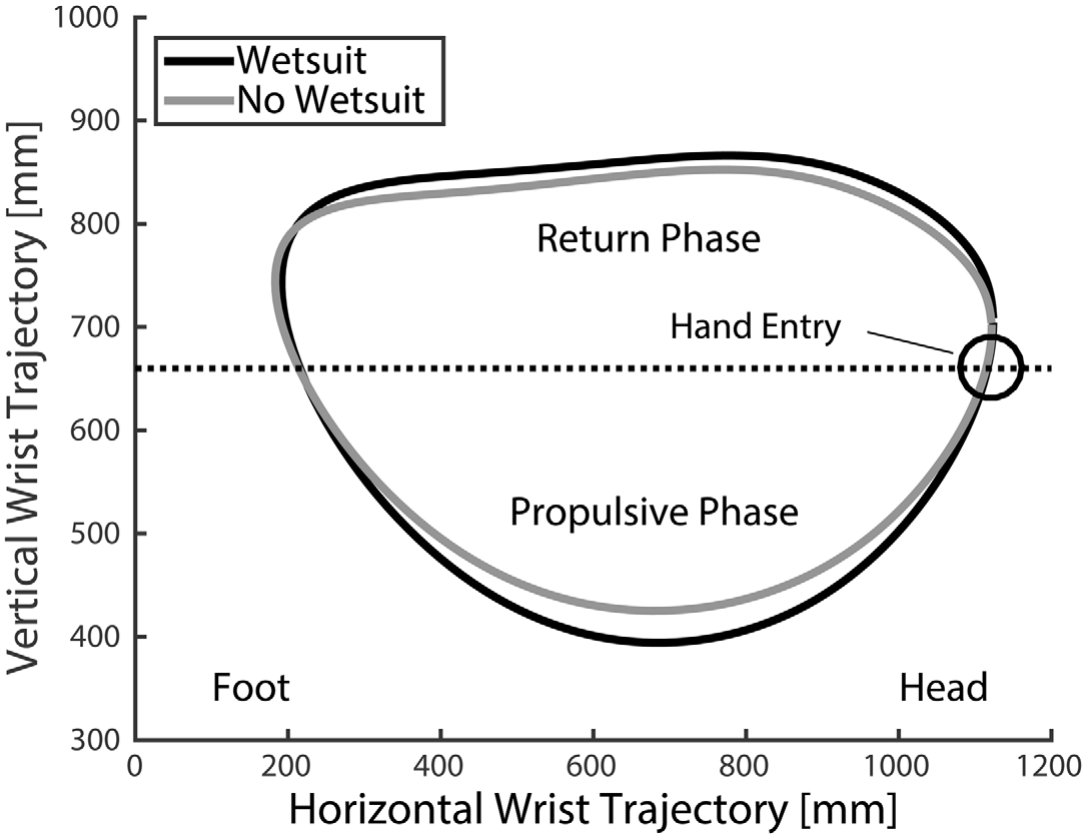
Mean sagittal plane trajectory for the right wrist with and without a wetsuit. Horizontal line represents and estimate for water level, though subjects paddled an ergometer in the absence of water. The point of greatest anterior (caudal) position of the hand determined the beginning of the stroke, and the propulsive and return phases occurred at approximately 20–70% (bottom of trajectory) and 70 to 20% (top of trajectory) of the stroke cycle, respectively.

Sagittal plane wrist trajectory during the paddling motion was significantly altered when subjects wore a wetsuit. In particular, the vertical range of motion of the wrist trajectory increased for the wetsuit condition (467.7±91.5mm vs 424.3±77.9mm, p = 0.019, Table 2, Fig 3). This change in vertical range of motion resulted primarily from subjects shifting the lowest point in their stroke upward: the minimum hand position per stroke reached significantly lower for the wetsuit condition when compared to the no wetsuit condition (401.5±57.5mm vs 432.1±52.4mm, p = 0.003) while the peak (upper) hand position per stroke was not different between conditions (869.2±65.1mm vs 856.3±44.0mm, p>0.05). No differences in average stroke length (970.0±64.2mm vs 971.6±76.9mm, p>0.05), stroke width (169.9±66.8mm vs 171.8±59.4mm, p>0.05), stroke duration (2.31±.3sec vs 2.28±0.3sec, p>0.05) or shoulder range of motion in the sagittal (136.8±30.8° vs 128.5±15.5°, p>0.05) and transverse planes (89.9±7.5° vs 91.8±13.8°, p>0.05) were found between conditions.

**Table 2:** Kinematic Results.

	Antero-posterior [Stroke Height]		Cranial-caudal [Stroke Length]		Medio-lateral [Stroke Width]	
**Condition**	**Wetsuit**	**NW**	**Wetsuit**	**NW**	**Wetsuit**	**NW**
**Mean Excursion [mm]**	467.7±91.5*	424.3±77.9	970.0±64.2	971.6±76.9	169.9±66.8	171.8±59.4
**λ**_**short**_ **(0–1 stroke)**	1.49±0.26	1.38±0.28*	1.93±0.25	1.91±0.19	1.04±0.30	1.03±0.18
**λ**_**long**_ **(10 strokes)**	0.035±0.036	0.027±0.029	0.047±0.044	0.041±0.030	0.022±0.024	0.021±0.023
**Approximate Entropy**	0.45±0.05*	0.39±0.09	0.46±0.13	0.43±0.11	0.50±0.11	0.48±0.09

Values are reported as mean±SD

* denotes p<0.05

### Approximate Entropy and Maximal Lyapunov Exponents

**Fig 4:**
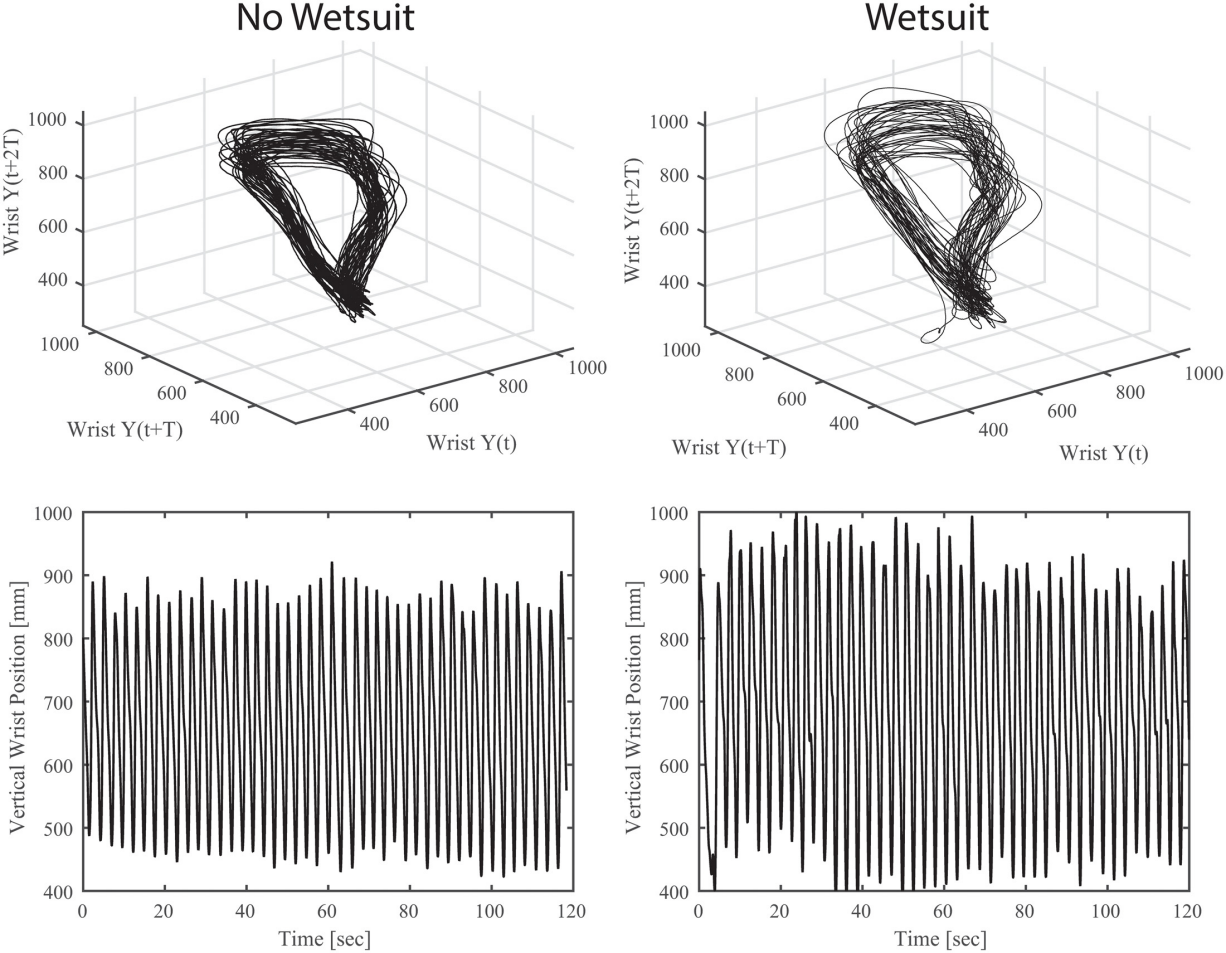
**Top: Three dimensional state space plots for a representative subject for wrist movement in the vertical direction.** An embedding delay of 20 was used. **Bottom: Raw wrist trajectory in the vertical direction.**

Wearing a wetsuit resulted in a significant increase in ApEn for vertical range of motion of the wrist trajectory in the sagittal plane (0.45±0.05 vs 0.39±0.09, p = 0.019, Table 2). Changes in entropy were not statistically significant for stroke length, stroke width, stroke duration, and shoulder range of motion in the sagittal or frontal planes. Wearing a wetsuit also resulted in a significant increase in short term maximal Lyapunov exponents for the vertical trajectory of the wrist (1.49±0.26 vs 1.38±0.28, p = 0.036, Table 2, Fig 4). Though average values for both short and long term LyE were consistently greater when subjects wore a wetsuit, no statistically significant differences were found for any other direction of wrist trajectory.

### Muscle Activity

**Fig 5:**
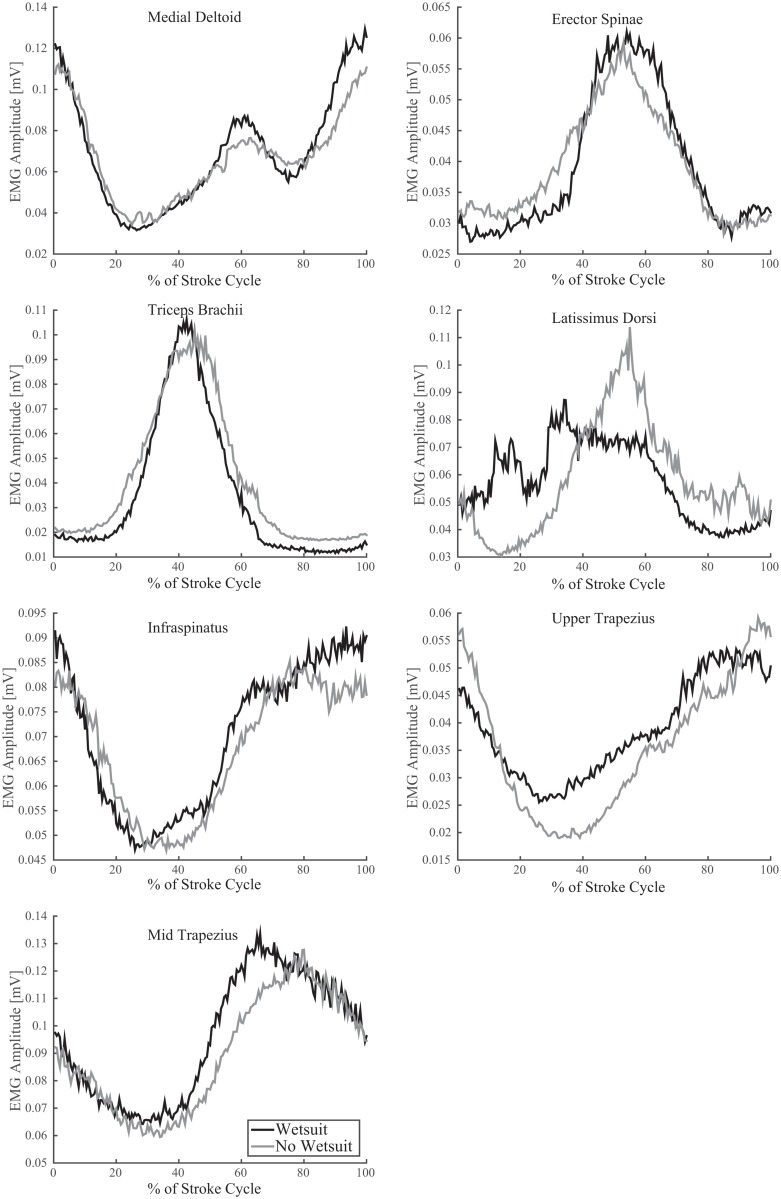
Mean EMG activation of select shoulder and trunk muscles throughout the paddling stroke. EMG data were rectified, filtered, and averaged across all complete strokes per subject, then across all subjects. The horizontal access defines the percentage of the stroke cycle (0% represents the beginning, 100% represents the end of the cycle). Grey lines represent the no wetsuit condition, while the black lines represent the wetsuit condition.

Wearing a wetsuit resulted in a significant increase in peak EMG activity for the middle deltoid (0.14±0.08 vs 0.11±0.05, p = 0.041, Table 3, Fig 5). No significant differences in patterns of muscle activation were found for *triceps brachii*, *infraspinatus*, *latissimus dorsi*, upper/mid *trapezius*, or *erector spinae* between the wetsuit and no wetsuit conditions. This comparison included peak activation, burst duration, and area under the curve (linear envelope) for each signal. High levels of motion artifact corrupted the signals from *pectoralis* major for several subjects due to periodic contact with the surfboard deck. Therefore these data were excluded from further analysis.

**Table 3:** Electromyography Results.

	Peak [mV]		Linear Envelope [mV*ms]		Burst Duration [% of stroke]	
Condition	Wetsuit	NW	Wetsuit	NW	Wetsuit	NW
*Triceps Brachii*	0.11±0.07	0.11±0.04	5.4±2.0	5.3±2.4	47.4±14.3	49.6±13.7
*Upper Trapezius*	0.06±0.03	0.06±0.03	5.6±2.5	4.9±2.5	79.0±11.1	74.8±11.4
*Mid Trapezius*	0.15±0.10	0.14±0.06	14.7±8.6	13.8±7.0	74.7±11.1	75.6±10.7
*Medial Deltoid*	0.14±0.08	0.11±0.05*	10.1±5.5	9.8±4.1	74.1±15.7	81.4±8.9
*Infraspinatus*	0.10±0.09	0.09±0.06	9.5±6.5	9.1±5.0	79.1±7.7	79.8±8.7
*Latissimus Dorsi*	0.12±0.09	0.13±0.08	8.2±4.2	8.6±5.8	58.6±17.3	58.0±16.1
*Erector Spinae*	0.06±0.03	0.06±0.02	5.9±2.2	5.7±2.3	73.2±17.2	79.5±14.1

Values are reported as mean±SD

* denotes p<0.05

### Metabolic and Thermal Effects

No differences were noted in oxygen consumption between the wetsuit and no wetsuit conditions (Table 4). Skin temperature was significantly higher during the wetsuit condition. All readings of workload and cadence were consistent between the wetsuit and no wetsuit trials and no subjects were excluded from the final analysis due to these variables.

**Table 4:** Physiological Measurements.

	Cadence [spm]	Workload [watts]	O^2^ Consumption [ml/kg/min]	Mean HR [bpm]	Mean Skin Temp [°C]
**Wetsuit**	25.2±2.7	20.2±1.9	14.2±1.2	113.8±11.7	34.4±0.9
**No Wetsuit**	26.1±3.4	20.6±1.6	14.2±1.8	114.2±18.4	33.1±1.3*

Values are reported as mean±SD

## Discussion

There were four primary results to this experiment. First, wearing a wetsuit resulted in a significant increase in complexity (ApEn) and movement variability (LyE) for vertical wrist trajectory. Second, wearing a wetsuit resulted in a significant increase in the peak activity of the middle deltoid, but changes in activity were not noted in any of the other muscles studied here. Third, while wearing a wetsuit did not result in any changes to shoulder range of motion, a significant increase in the average vertical range of motion of the sagittal plane wrist trajectory was noted. Finally, wearing a wetsuit did not result in any increase in oxygen consumption or heart rate for simulated surfboard paddling at a constant velocity. Taken together, these results suggest that when paddling at this particular rate (25 strokes/min) and intensity (20 Watts), wearing a wetsuit does not result in any increase in metabolic cost but does contribute to changes in muscle activity and paddling motion. These results support the hypothesis that a wetsuit may provide enhanced proprioceptive acuity, but do not support the hypothesis that a wetsuit provides increased resistance to the paddling motion.

### Muscle Activation

The EMG data demonstrate that muscle activation about the shoulder during the paddling motion can be divided broadly into muscles that either contribute to 1) propulsion of the body or 2) the return phase of the hand (i.e. scapular retraction and horizontal abduction and flexion of the shoulder). Previous researchers investigating the swimming stroke have used the analogous terms of “pull-through” and “recovery” [60]. In the current analysis, the greatest anterior (caudal) position of the hand was defined as the beginning of the stroke (Fig 3). Therefore, the propulsive phase corresponded roughly to the region bounded by 20 to 70% of the stroke duration, while the return phase was bounded roughly by 70 to 20% of the cycle, with a theoretical “hand entry” likely occurring around 0–20% of the paddling stroke.

In the current EMG data, *latissimus dorsi* and *triceps brachii* experienced peak activation at around 40 and 50% of the stroke cycle (i.e. mid-propulsive phase), respectively, suggesting that these muscles contributed primarily to propulsion (Fig 4). In addition, *erector spinae* exhibited peak activation during the mid-propulsive phase of the stroke, suggesting that its primary role in the paddling motion is to provide stability for forceful shoulder and elbow extension on the ipsilateral side. The remaining muscles, *infraspinatus*, mid and upper *trapezius*, and middle *deltoid*, were most active at around 80–100% of the cycle, or during the middle of the return phase. Upper *trapezius* and *infraspinatus* remained active longer than mid *trapezius*, suggesting that its contribution may occur primarily during the middle of the return phase. Middle *deltoid* was most active late in the return phase, but also exhibited a smaller peak at approximately 60% of the stroke, suggesting that it may also play a role in augmenting the propulsive activity of *latissimus dorsi* and *triceps brachii*. In general, these results are comparable to those obtained previously during free-style swimming, particularly the unique activation pattern of the middle *deltoid* [60,61]. While this analysis provides an introductory layout of muscle activation surrounding the shoulder joint during surfboard paddling, additional study will be necessary to discern activation patterns in greater detail, as well as the role of other muscles including, *supraspinatus*, *subscapularis*, *serratus* anterior, and *pectoralis* major.

### Attractor Dynamics and Motor Learning

Many studies in motor learning are focused on the attractor dynamics that represent the output of a motor system (e.g. [18,32,62,63]). This idea developed in part from Bernstein’s degree of freedom problem [64] together with a more recent distinction between physiological degrees of freedom and the dimension of the attractor dynamic (e.g. [65]). An attractor can be defined as the solution or set of solutions to a particular movement problem toward which a system tends to evolve. When perturbed slightly, a person’s performance remains in the neighborhood of their behavioral attractor for that particular skill and typically does not deviate to a large degree. The attractor dynamic is inherently complex because it integrates physiological degrees of freedom, the coupling constraints between degrees of freedom, and the behavior of feedback loops that operate on different time scales.

Approximate entropy (ApEn) provides a measure of the complexity of the output of a motor system, which is often associated with the dimension of the attractor dynamic [65]. In the current data, wearing a wetsuit resulted in an increase in the complexity of the vertical range of motion, which has previously been associated with proficiency or learning a new motor skill [38,39]. However, a decrease in complexity has also been associated with learning a motor skill, suggesting that the direction of change in response to practice (increase vs decrease) can vary and is unique to the task being learned [62,66].

The surfers utilized in the present study were all proficient paddlers. Therefore, the observed change in complexity is likely not attributable to practice effects, but rather a change in control strategy which could have been induced by the presence of additional sensory information. While there is little direct evidence to support this theory, evidence does appear to exist for the converse; individuals with impaired sensory input have been shown to demonstrate a decrease in movement related complexity [35,36]. In addition, there is evidence to support the idea that a neoprene sleeve can provide additional proprioceptive input [10–12]. Both of these ideas support the theory that enhanced proprioceptive input contributed to the observed changes in ApEn observed here.

Estimates of maximal Lyapunov exponents can provide a measure of variability or attractor divergence for a motor system [30]. Lower values of LyE represent a system that stays in the neighborhood of the attractor when perturbed slightly and converges toward the attractor relatively quickly as time is incremented forward. This concept is often referred to as local dynamic stability, and is related to the overall variability of the output of a system [29]. Fig 4 depicts a graphical representation of the attractor for wrist movement in the Y direction in which a noticeable increase in variability of the state-space representation (i.e. higher LyE) is present for the wetsuit condition. Previous research suggests that there is an optimal level of movement variability that is not too low (i.e. inflexible) or too great (noisy) [33]. Without knowledge of a “normal” or baseline level of variability it is difficult to determine if this increase is beneficial to the paddling athlete. However, in light of the observations that 1) the subjects included here were proficient paddlers and not impaired or prone to injury, and 2) the addition of a neoprene sleeve is more likely to enhance proprioceptive feedback rather than diminish it [13], the more likely interpretation is that the observed increase in variability represents a benefit to paddling performance.

### Energy Expenditure

The metabolic data demonstrate that wearing a wetsuit while paddling at this rate and intensity had little effect on oxygen consumption and heart rate. Similarly, changes in muscle activation were only noted for one muscle (middle *deltoid*) when a wetsuit was worn. These data suggest that the changes in paddling motion observed here are likely not related to changes in energy cost or effort associated with wearing a wetsuit. Further, because the order of wetsuit condition was randomized across subjects and trial length was kept relatively short (5 minutes) in experienced surfers, it is unlikely that muscular fatigue contributed to the observed differences in paddling motion. Finally, while these data suggest that a wetsuit has little effect on paddling energy cost and effort at this particular velocity, it is possible that a metabolic effect might be found at different paddling rates and intensities.

## Limitations

These results were generated while paddling an ergometer in the laboratory and wearing a dry wetsuit. There are a number of differences between paddling in water and on the ergometer, as well as differences in the behavior of the wetsuit itself when water is present. First, the surface of the water provides a spatial reference point in the vertical direction that an athlete might use during the paddling motion. No such reference point was present in the current study, though subjects were asked to raise their hands above an imagined surface of the water with each stroke. Second, physical interaction with water itself may provide additional sensory feedback, including proprioceptive information, and this may also contribute to alterations in the paddling motion. The paddling ergometer was therefore appropriate for an initial analysis because it allowed for isolation of the proprioceptive effects of wearing a wetsuit from interaction with water. Third, the drag force experienced during the propulsive phase of the paddling stroke in water is different from the resistance generated by the ergometer. Finally, retention of water in the neoprene itself can change the material properties of the wetsuit, which may also lead to changes in paddling behavior. In particular, the increased mass of added water may increase resistance to movement, and water retention may alter the elastic properties of the neoprene. Further research is necessary to determine how the current results can be extended to paddling in water.

The nonlinear analyses techniques utilized here do not represent the only approaches available for examining the complex, nonlinear behavior of human movement. It is possible that other approaches may yield differing results or offer additional insight for the paddling motion. For example, estimation of the embedding dimension (*d*_*E*_) and embedding delay (*τ*) of wrist trajectory for phase-space reconstruction might have been accomplished using an approach that assumes that these two parameters are related [52,53,57]. If one of these analyses yielded values for *d*_*E*_ and *τ* that differed from those used in the current study, it is likely that the analysis of maximum Lyapunov exponents would have also generated a different result. Further, calculating Lyapunov exponents to examine local dynamic stability of upper extremity motion yielded interesting results, but analysis techniques from complex network theory are likely more robust to noise and may provide additional information. This approach would involve mapping time series data onto a functional network that represents the structural interrelationships among data in the original series [22,24]. Mapping the data in this manner allows for more detailed evaluation of the connectivity and topological properties of this complex system. This approach has been shown to be useful in the analysis of systems with a large number of components that interact in a complicated manner [19–23,25,67,68]. Specific examples of this approach include directed weighted complex network (DWCN) analysis [20], and recurrence quantification analysis [25].

## Conclusions

Overall, these data suggest that wearing a wetsuit may enhance an athlete’s paddling technique. In particular, the trajectory of the wrist became more complex and increased in variability when a wetsuit was worn. Previous literature suggests that these changes may be the result of the compressive effect of a wetsuit and its effect on proprioceptive acuity [10–13]. These results should be considered in the design of wetsuits or garments for surfers that can be worn in warmer conditions that provide proprioceptive stimulus without thermoregulatory influence. In addition, these findings may have implications for other aspects of movement in the upper extremity, including those performed by overhead athletes and individuals recovering from neurological injury.
